# Characteristic mTOR activity in Hodgkin-lymphomas offers a potential therapeutic target in high risk disease – a combined tissue microarray, in vitro and in vivo study

**DOI:** 10.1186/1471-2407-13-250

**Published:** 2013-05-22

**Authors:** Ágnes Márk, Melinda Hajdu, Zsófia Váradi, Tamás Béla Sticz, Noémi Nagy, Judit Csomor, Lajos Berczi, Viktória Varga, Monika Csóka, László Kopper, Anna Sebestyén

**Affiliations:** 1 1st Department of Pathology and Experimental Cancer Research, Semmelweis University, Üllői út 26, Budapest 1085, Hungary; 2 2nd Department of Pediatrics, Semmelweis University, Tűzoltó u. 7-9, Budapest 1094, Hungary; 3 Tumor Progression Research Group of Joint Research Organization of the Hungarian Academy of Sciences and Semmelweis University, Budapest, Hungary

**Keywords:** mTOR activity, Hodgkin-lymphoma, Rapalogs, TMA, Hodgkin-lymphoma xenograft

## Abstract

**Background:**

Targeting signaling pathways is an attractive approach in many malignancies. The PI3K/Akt/mTOR pathway is activated in a number of human neoplasms, accompanied by lower overall and/or disease free survival. mTOR kinase inhibitors have been introduced in the therapy of renal cell carcinoma and mantle cell lymphoma, and several trials are currently underway. However, the pathological characterization of mTOR activity in lymphomas is still incomplete.

**Methods:**

mTOR activity and the elements of mTOR complexes were investigated by immunohistochemistry on tissue microarrays representing different human non-Hodgkin-lymphomas (81 cases) and Hodgkin-lymphomas (87 cases). The expression of phospho-mTOR, phospho-4EBP1, phospho-p70S6K, phospho-S6, Rictor, Raptor and Bcl-2, Bcl-xL, Survivin and NF-kappaB-p50 were evaluated, and mTOR activity was statistically analyzed along with 5-year survival data. The in vitro and in vivo effect of the mTOR inhibitor rapamycin was also examined in human Hodgkin-lymphoma cell lines.

**Results:**

The majority (>50%) of mantle cell lymphoma, Burkitt lymphoma, diffuse large B-cell lymphoma, anaplastic large-cell lymphoma and Hodgkin-lymphoma cases showed higher mTOR activity compared to normal lymphoid tissues. Hodgkin-lymphoma was characterized by high mTOR activity in 93% of the cases, and Bcl-xL and NF-kappaB expression correlated with this mTOR activity. High mTOR activity was observed in the case of both favorable and unfavorable clinical response. Low mTOR activity was accompanied by complete remission and at least 5-year disease free survival in Hodgkin-lymphoma patients. However, statistical analysis did not identify correlation beetween mTOR activity and different clinical data of HL patients, such as survival. We also found that Rictor (mTORC2) was not overexpressed in Hodgkin-lymphoma biopsies and cell lines. Rapamycin inhibited proliferation and induced apoptosis in Hodgkin-lymphoma cells both in vitro and in vivo, moreover, it increased the apoptotic effect of chemotherapeutic agents.

**Conclusions:**

Targeting mTOR activity may be a potential therapeutic tool in lymphomas. The presence of mTOR activity probably indicates that the inclusion of mTOR inhibition in the therapy of Hodgkin-lymphomas may be feasible and beneficial, especially when standard protocols are ineffective, and it may also allow dose reduction in order to decrease late treatment toxicity. Most likely, the combination of mTOR inhibitors with other agents will offer the highest efficiency for achieving the best clinical response.

## Background

The number of patients diagnosed with lymphoid malignancies has increased to 18,000 per year in Europe [1]. Hodgkin-lymphomas (HL) with characteristic histopathological subtypes comprise about 11% of all lymphomas [1,2]. Tumor cells [Hodgkin-/Reed-Sternberg (HRS) cells] usually represent only a small fraction of diagnostic histology, while differences in microenvironment (reactive lymphocytes, extracellular matrix) allow subclassification of HL [3,4]. The prognosis of HL patients is relatively good, however, some patients may relapse in spite of first line chemotherapy and radiation protocols, and can be further treated, sometimes cured by intensified chemotherapy and/or peripheral stem cell transplantation [5]. Unfortunately, these treatments still fail in 15-20% of HL patients [6]. Considering that the majority of HL patients are young and the survivors have a high risk of acute or late toxicity associated with therapy [7], more efficient and less toxic therapeutic strategies are needed. Targeting signaling pathways offers an attractive approach.

The PI3K/Akt/mTOR pathway is activated in a number of human neoplasms, accompanied by lower overall and disease free survival [8]. This pathway plays a key role in the regulation of cellular functions such as survival, proliferation, cell death and metabolic activities [9]. mTOR (mammalian target of rapamycin) – an important component of this network – is a serine-threonine kinase, which exists in two distinct multiprotein complexes (mTORC1 and mTORC2 – containing characteristic elements: Raptor and Rictor, respectively) [10]. The best known targets of mTORC1 are eukaryotic initiating factor-4E binding proteins (4EBP) and S6 kinase (S6K). mTORC2 can regulate Akt dependent antiapoptotic and survival mechanisms by phosphorylating Akt [11].

The PI3K pathway can be activated by several upstream receptors (IGF-R, Flt3, c-Kit, Notch, TCR, BCR) or intracellular proteins (Ras, BCR/ABL) in various hematological diseases [12]. Information about mTOR activity is very limited; however, transforming direct genetic modifications of PI3K, Akt, mTOR or PTEN are rare – such mutations occur in 5% of lymphoid malignancies [13]. mTOR has indeed been proven an important element in tumorigenesis in mantle cell lymphoma (MCL): its role was confirmed in MCL cell proliferation, mainly by influencing cyclin D1 expression [14]. This suggests that the mTOR pathway may play an important role in the development or progression of other lymphoma types as well, and can be considered as a useful therapeutic target.

Rapamycin (and its analogs: rapalogs) interacts with the FKBP12 protein, an element of the mTOR complex, and preferentially disrupts mTORC1 activity [15]. The response of mTORC2 to rapalogs remains conflicting [16]. Rapalogs have been used as immunosuppressive agents in organ transplantation since 1999, and they have been introduced into clinical oncology as a treatement option in renal cell carcinoma and recently in MCL as well [14]. Several trials using mTOR inhibitors in tumors with high mTOR activity are currently underway [17-19].

The aim of our study was to investigate mTOR activity in different lymphomas, with a focus on HL. We found that the majority of HL cases (93%) displays high mTOR activity. Therefore we suggest that mTOR inhibition (e.g. by rapalogs) may be considered as a therapeutic option in HL, especially in patients with poor prognosis/relapse.

## Methods

### Cell culture

KM-H2, L428, L1236, HDLM2, DEV (Hodgkin-lymphoma) cell lines were cultured in RPMI 1640 supplemented with 100 U/ml penicillin, 100 ng/ml streptomycin (Sigma) and heat-inactivated 10% FCS (Gibco). The UH-01 (HL) cell line was cultured in Iscove's MDM + RPMI-1640 (4:1) supplemented with 20% FCS, 2 mM L-glutamine (Sigma) and penicillin and streptomycin as above.

Cells were treated with rapamycin (50 ng/ml, Sigma) for 72 h; culture medium was refreshed with new medium supplemented with rapamycin after 72 h to avoid rapamycin concentration decrease (due to metabolic degradation) in longer treatments (96–144 h). Combination treatments in HL cell lines were done for 72 hours. Doxorubicin (0.2 μM; Ebewe Pharma), vincristine (10 nM; Richter Gedeon) and etoposide (1 μM; Pharmachemie BV) were used in combination with rapamycin. Cell morphology was evaluated on methanol fixed and hematoxylin-eosin (HE) stained cytospin preparates.

### Western-blotting

Whole cell extracts were prepared and quantitated with Quant-iT protein assay (Invitrogene). Protein extracts (112.5 μg) were transferred to PVDF membranes after SDS-PAGE. Membranes were incubated with anti-phospho-mTOR (Ser2448), anti-mTOR, anti-phospho-p70S6K (Thr389) and anti-phospho-S6 (Ser235/236) antibodies (Cell Signaling), followed by biotinylated secondary antibodies and avidin-HRP complex (Vectastain Elite ABC Kit, Vector), and detected by enhanced chemiluminescence (Pierce ECL Western Blotting Substrate). Membranes were stripped (Re-Blot Plus, Millipore) and reprobed with β-actin (A2228; Sigma) to confirm equal protein loading.

### Enzyme-linked immunosorbent assay (ELISA)

Cell lysates were obtained from isolated normal B- and T-cells, normal mononuclear cells from buffy coat and lymphoma/leukemia cell lines (5×10^6^ cells/sample) in lysis buffer (Cell Signaling) containing 1 mM phenyl-methylsulfonyl fluoride (PMSF) for 30 minutes on ice. Sandwich ELISA Kit (p4EBP1 – Thr37/Thr46, Cell Signaling) was used for the detection of phospho-4EBP1 according to the manufacturer’s instructions. Optical density (OD) was measured at 450 nm wavelength.

### Flow cytometry

For apoptosis detection cells were fixed in 70% ethanol (−20°C) followed by alkalic extraction (200 mM Na_2_HPO_4_, pH 7.4 and 100 mg/ml RNase; Sigma) and propidium-iodide staining (1 mg/ml, Sigma) according to Mihalik et al. [20]. A minimum of 10,000 events/sample were acquired on a FACScan flow cytometer (BD Biosciences, Erembodegem, Belgium). Data were analyzed with WinList software (Verity Software House, Topsman, ME, USA).

### Tissue microarray (TMA) and Hodgkin-lymphoma patients

Formalin-fixed paraffin-embedded biopsy specimens from 105 lymphoma patients (6 Burkitt-lymphomas [BL], 23 HL, 11 MCL, 9 anaplastic large-cell lymphomas [ALCL], 9 diffuse large B-cell lymphomas [DLBCL], 12 marginal zone lymphomas [MZL], 13 chronic lymphoid leukemias/small lymphocytic lymphomas [CLL], 10 follicular lymphomas, 12 peripheral T-cell lymphomas) were included in the first TMA study. The total number of HL patients was 83 in the second TMA set, which represented all HL subtypes: nodular lymphocyte predominant (NLPHL) and classical HL (cHL) types (7 and 76 cases, respectively). cHL samples included nodular sclerosis (n=47), mixed cellularity (n=18), lymphocyte rich (n=8) and lymphocyte depleted (n=3) cases. In each case, two representative cores of 2 mm diameter were selected from different areas. Reactive lymphoid tissues (tonsils and lymph nodes) were also included as non-neoplastic controls.

Hodgkin-lymphoma patients (40 females, 43 males; age: 8–82 years [23 patients<18 years, 41 patients: 18–45 years, 15 patients: >45 years]; mean age: 29.8 years) were diagnosed at our Institute between 2000 and 2007. The minimum follow-up period was 5 years in all cases. Clinical data were available in detail in 72 cases from the analyzed 83 patients: 59 of these patients were in complete remission after 5 years of follow-up, 25 patients had relapse and 10 patients died, 13 patients had stem cell transplantation. 60% of these relapsed patients (15/25) are now in CR, including 8 patients who achieved CR following stem cell transplantation. The majority (64%) of the patients had stage I-II disease, whereas 36% presented with stage III-IV disease; 30% of the patients had B-symptoms.

For pediatric and adolescent patients (8–18 years), treatment group (TG) 1 (stages IA/B, IIA) received 2 cycles OPPA (females) or OEPA (males); TG2 (stages IIB, IIIA, I_E_A/B, II_E_A) received 2 cycles OPPA or OEPA and 2 cycles COPP; TG3 (IIIB, IVA/B, II_E_B, III_E_A/B) received 2 cycles OPPA or OEPA and 4 cycles COPP. Additional radiotherapy and/or autologous/allogeneic hematopoietic stem cell transplantation (HSCT) was given in the case of incomplete remission. (OPPA: vincristine, procarbazine, prednisone, doxorubicin; OEPA: vincristine, etoposide, prednisone, doxorubicin; COPP: cyclophosphamid, vincristine, procarbazine, prednisone).

Adult patients were treated with ABVD; DHAP protocol was used in the case of ABVD-resistance. DHAP was also given before HSCT. (ABVD: adriamycin, bleomycin, vinblastine, dacarbazine; DHAP: dexamethasone, high dose cytarabine, cisplatin).

All protocols were approved by the Institutional Ethical Review Board (TUKEB no. 7/2006).

### Immunocytochemistry/Immunohistochemistry (ICC/IHC)

Four μm TMA sections were deparaffinized. Endogenous peroxidase blocking was followed by antigen retrieval in sodium citrate (pH=6) buffer in a microwave oven.

Cytospin preparates were fixed in 80% methanol (10’,-20°C), and incubated with primary antibodies following endogenous peroxidase blocking.

Slides were incubated overnight at 4°C with phospho-S6 (Ser235/236), phospho-mTOR (Ser2448), phospho-4EBP1 (Thr37/46), phospho-p70S6K (Thr389), phospho-Histone-H3 (pHH3), cleaved/activated caspase3 (Cell Signaling), Rictor (Abcam), Raptor (Novus), CD15 (Leica), CD30, MUM-1, Bcl-xL, Bcl-2 (Dako), NF-kappaB-p50 and Survivin (LabVision) antibodies.

Primary antibodies were followed by Novolink Polymer Detection System (Novocastra, Wetzlar, Germany), visualized by DAB and counterstained with hematoxylin. Immunostainings were evaluated by 2 independent pathologists. 3DHistech Pannoramic Viewer program and Nikon E200 were used for tissue microarray analysis.

Phospho-mTOR, phospho-4EBP1, phospho-p70S6K, phospho-S6 TMA immunostaining reaction intensity (negative, 1+(weak)/2+(moderate)/3+(strong) positive) was agreed upon before blind evaluation of the scores (0/1+/2+/3+). Non malignant, reactive lymphocytes showed a maximum positivity of 1+, whereas plasma cells were score 3+.

The most reliable phospho-protein marker for mTOR activity was phospho-S6, which is supported by literature data. Therefore, the cases in our study were considered to have high mTOR activity only when scores were 2+/3+ for phospho-S6 and for at least one additional mTOR activity related phosphoprotein (pmTOR, pp70S6K), as described previously [21].

NF-kappaB-p50 was considered positive when nuclear staining was observed; Bcl-2 and Bcl-xL positivity was cytoplasmic. Survivin showed both nuclear and cytoplasmic positivity.

The cutoff for positivity was set at 10% of the tumor cells staining for the antibodies, according to Sebestyén et al. [21].

### Hodgkin-lymphoma xenograft model

Xenograft tumors were established in SCID mice by injecting 2×10^7^ KMH2 cells subcutaneously (s.c.) with matrigel into the back region of 8–10 week old (20–23 g) mice. Palpable tumors were removed, cut into pieces and transplanted into secondary recipient mice. When palpable s.c. tumors developed (after 8 weeks), animals were divided into control and rapamycin-treated groups (n=10 each). Rapamycin (Rapamune 1 mg/ml, Wyeth Europa Ltd.) was administered by gavage at 3 mg/kg body weight three times per week for 8 weeks. Control groups were treated with saline. Body weight and tumor diameter was measured weekly. Tumor volume was calculated as follows: п/6×(2×shorter diameter + longer diameter)/3)^3^. Tumor weight was measured in euthanized animals at the end of the experiments. Tumor tissues were formalin-fixed, paraffin-embedded and immunostained with human CD15, human CD30, cleaved/activated caspase3 and pHH3. pHH3 and cleaved/activated caspase3 stainings were analyzed with Mirax Viewer software (analysing 4 areas in each sample).

All experiments involving laboratory animals were done in accordance with the Guidelines for Animal Experiments of the Office of Agricultural Administration of Budapest and by the Animal Research Comittee of our university (permission number: 201/2010).

### Statistics

Statistics was calculated with paired Student’s t-test, Chi square test and Fisher’exact test using SPSS (SPSS Inc., Chicago, IL, USA) and PAST softwares (PAST free software was downloaded from http://folk.uio.no), and log-rank test using GraphPad software (GraphPad, San Diego, California, USA).

## Results

### mTOR activity is increased in lymphoma cells

**Figure 1:**
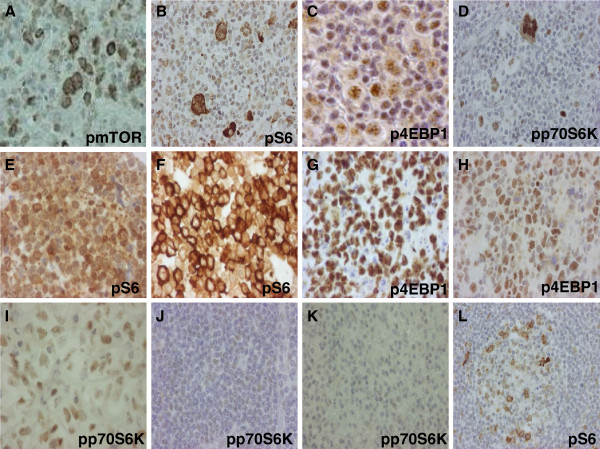
**mTOR activity is increased in lymphoma cells.** mTOR activity related phosphoproteins in different lymphomas detected by IHC. Lymphomas with high mTOR activity (2+/3+): Hodgkin-lymphoma (**A-D**), mantle cell lymphoma (**E**), Burkitt-lymphoma (**F-G**), diffuse large B-cell lymphoma (**H**), anaplastic large-cell lymphoma (**I**). Lymphomas with low mTOR activity (0/1+) comparable to non malignant lymphoid cells: chronic lymphoid leukemia/small lymphocytic lymphoma (**J**), marginal zone lymphoma (**K**); control lymph node (**L**); (IHC), 200X, 400X.

mTOR activity was estimated by immunohistochemistry (IHC) with antibodies against the active form of mTOR and its target proteins on tissue microarray (TMA) sections representing different lymphomas. The evaluation of the mTOR activity stainings of lymphoma subtypes showed high mTOR activity in the majority (>50%) of mantle cell lymphoma (11/11), Burkitt-lymphoma (6/6), diffuse large B-cell lymphoma (5/9), anaplastic large-cell lymphoma (8/9) and Hodgkin-lymphoma cases (23/23). Compared to normal lymphoid tissues, HRS cells showed 2+/3+ positivity in virtually all Hodgkin-lymphoma samples in this first TMA study set (containing a limited number of cases). Regarding the analyzed cases of other lymphoma types, no or only low (0/+) mTOR activity was detected in marginal zone lymphomas, chronic lymphoid leukemias/small lymphocytic lymphomas and peripheral T-cell lymphomas (8/12, 12/13 and 10/12 negative/low, respectively; Figure 1). IHC results were conflicting in follicular lymphoma cases, because 7/10 samples were positive for pmTOR, and 6/10 were positive for pp70S6K, but all samples were negative for pS6.

### Hodgkin-lymphoma is characterized by high mTOR activity

**Figure 2:**
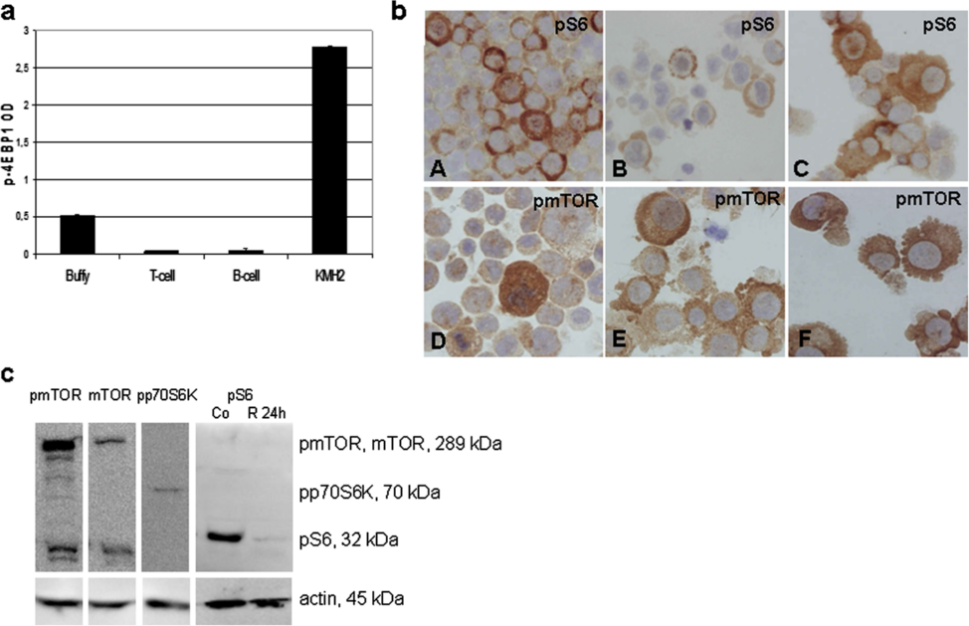
**mTOR signaling activity is increased in Hodgkin-lymphoma cell lines. a**. The amount of phosphorylated 4EBP1 protein is elevated in KMH2 Hodgkin-lymphoma cell line compared to normal B-cells, T-cells and buffy coat samples. (ELISA; p<0.05) **b**. Confirmation of mTOR activity in HL cell lines (KMH2, L428, UH-01, DEV, L1236, HDML2) by pS6 (A-C) and pmTOR (D-F); ICC (400X). **c**. mTOR kinase and phosphorylated proteins related to its activity (pmTOR, pp70S6K and pS6) in Hodgkin-lymphoma cells detected by Western-blotting. mTOR activity is rapamycin-sensitive in HL cell lines (Co: control; R: rapamycin-treated cells). Representative results showed in KMH2 HL cell line.

HL cell lines – KMH2, UH-01, L428, L1236, HDLM2 and DEV – showed high mTOR activity by ICC (Figure 2b). ICC results were confirmed by both Western-blotting and ELISA in KMH2 cells, and either Western-blotting or ELISA was performed in the other cell lines as well (Figure 2).

**Figure 3:**
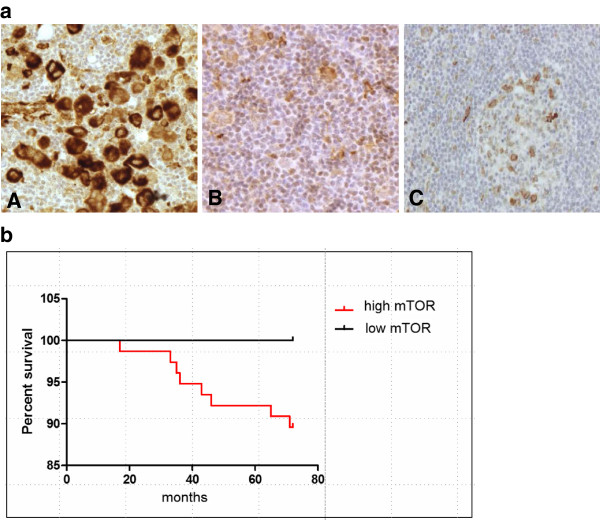
**mTOR activity in human Hodgkin-lymphoma biopsies. a**. High (A) and low (B) mTOR activity in lymphoma cells of Hodgkin-lymphoma biopsies. Low mTOR activity is comparable to that of reactive lymph nodes (C). (pS6 IHC; representative examples are shown.) **b**. Kaplan-Meier survival curves stratified by mTOR activity: low mTOR and high mTOR groups include 6 and 77 cases respectively (p=0.42).

A second set of TMA was constructed containing biopsy specimens from 83 HL patients. High mTOR activity was confirmed as a characteristic feature of HL (77/83), independently from the subtypes (NS: 44/47, MC: 17/18, LR: 8/8, LD: 3/3, NLPHL: 5/7) (Figure 3a). Non-malignant lymphoid tissues (tumor infiltrating lymphocytes, reactive tonsils and lymph nodes) showed low expression (0/1+) of mTOR-related phospho-proteins. IHC results were compared to the clinical data from 72 patients with long-term (a minimum of five-year) follow-up; we did not find a significant correlation with age, gender, stage, prognosis and histopathological type. We observed a tendency of correlation with therapeutic response and the present status of patients, but it did not reach statistical significance (p=0.42) (Figure 3b). It should be mentioned that all cases with low mTOR activity (6/72) were in complete remission with at least 5-year disease-free survival. Moreover, high mTOR activity (2+/3+) was detected in the biopsies of all patients who had poor prognosis and died (11 /72). However, high mTOR activity was observed in the case of both favorable and unfavorable clinical response.

**Figure 4:**
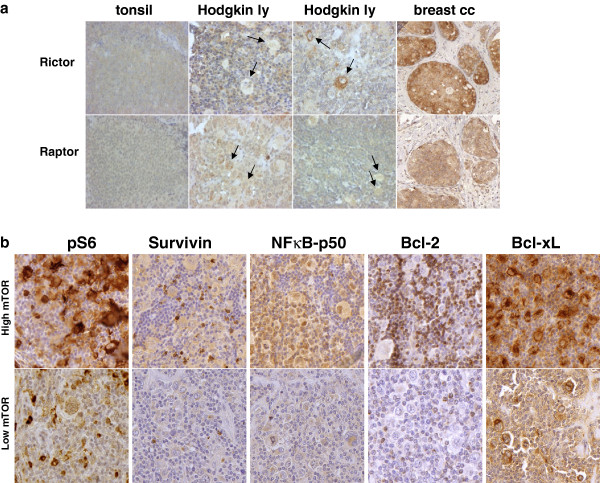
**Analysis of mTOR related protein expression in HL. a**. Hodgkin-lymphomas with no Rictor overexpression. Rictor and Raptor expression in HRS cells is similar to reactive lymphocytes (0/1+). Rictor expression is 0/1+ in the majority of HL cases (a representative example is shown); Rictor expression was high only in one HL sample (also shown here; arrows indicate tumor cells). Breast cancer cells show Rictor overexpression (2+/3+). (IHC, 200X, 400X). **b**. NF-kappaB-p50 and Bcl-xL expression may corralete to mTOR activity in HLs based on several IHC stainings; however, this tendency did not reach statistical significance (see results). (Representative IHC stainings are shown in cases with high and low mTOR activity; 400X).

We found that the expression of Raptor and Rictor (characteristic proteins of mTORC1 and mTORC2, respectively) by IHC was similar to the expression pattern of normal lymphocytes in 82 HL cases (Figure 4a). Rictor overexpression (2+/3+) (which was detected in several control breast carcinomas, indicating potential mTORC2 dominant expression) was detected only in one HL case.

Anti-apoptotic proteins (Bcl-2, Bcl-xL, Survivin and NF-kappaB-p50) known to be overexpressed in HLs were analyzed to search for a potential correlation and the role of mTOR activity behind their expression in HL (Figure 4b). High Bcl-xL expression was seen in the cytoplasm of HRS cells in all cases. NF-kappaB-p50 was expressed in 70% of HRS cells. 30% and 65% of the analyzed HL cases showed Bcl-2 and Survivin expression, respectively, which was significantly lower than the number of mTOR active cases. Based on these results, Bcl-xL and NF-kappaB-p50 expression may correlate with mTOR activity in HLs, but we did not find significance with Fisher’s exact test (p=0.07 and p=0.86, respectively); however, statistical analysis was hampered by the low number of cases with low mTOR activity.

### mTOR activity can be targeted in HL cells, leading to growth inhibition in vitro and in vivo

**Figure 5:**
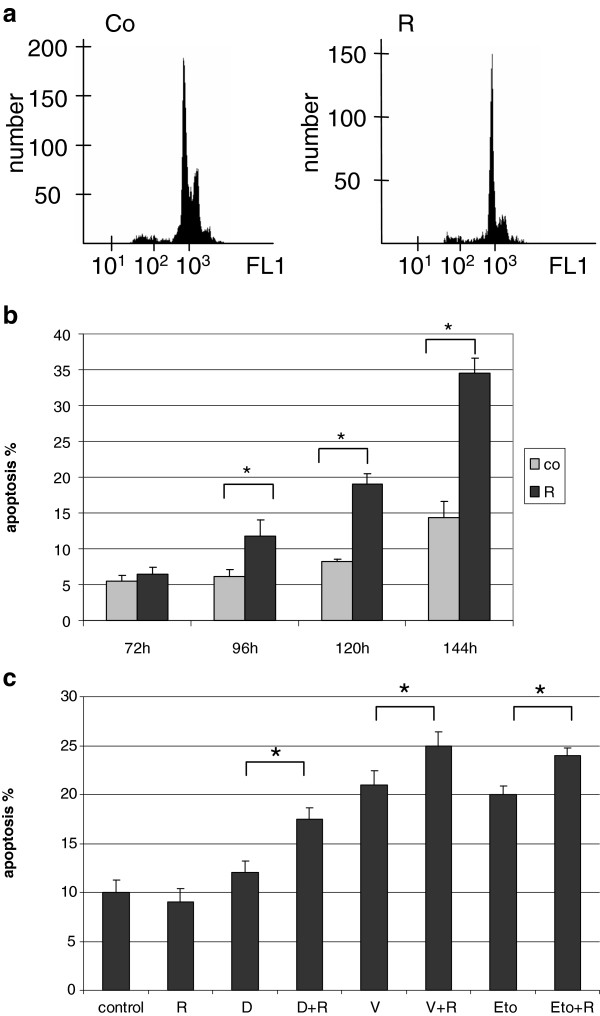
**Anti-proliferative and apoptotic effects of rapamycin in vitro. a**. G1 cell cycle arrest detected by flow cytometry after 72 h rapamycin treatment (50 ng/ml) in KMH2 Hodgkin-lymphoma cells. **b**. The apoptotic effect of rapamycin is time dependent in KMH2 cells (flow cytometry, *:p<0.05). **c**. Rapamycin treatment increased the apoptotic effect of chemotherapeutic agents in HL cell lines after 24 h. A representative experiment in KMH2 cells is shown here. (R: rapamycin 50 ng/ml, D: doxorubicin 0.2 μM, V: vincristine 10 nM, Eto: etoposide 1 μM; *:p<0.05).

Rapamycin treatment lead to G1 cell cycle block in all HL lymphoma cell lines without apoptosis induction after 72 h (Figure 5a). However, a longer (96-144 h) in vitro rapamycin treatment was able to switch on the apoptotic program (Figure 5b). The level of phosphorylated S6 was remarkably decreased, further supporting the inhibition of mTOR activity in HL cell lines (Figure 2c).

We investigated the effect of rapamycin combined with chemotherapeutic agents in KMH2, DEV and L1236 HL cell lines. When given in combination, rapamycin significantly increased the apoptotic effect of low dose “traditional” chemotherapeutic agents (doxorubicin, vincristine and etoposide) in KMH2 and DEV cell lines (Figure 5c). Rapamycin treatment had only an antiproliferative effect in L1236 cells, and could not enhance apoptosis induced by chemotherapeutic agents.

**Figure 6:**
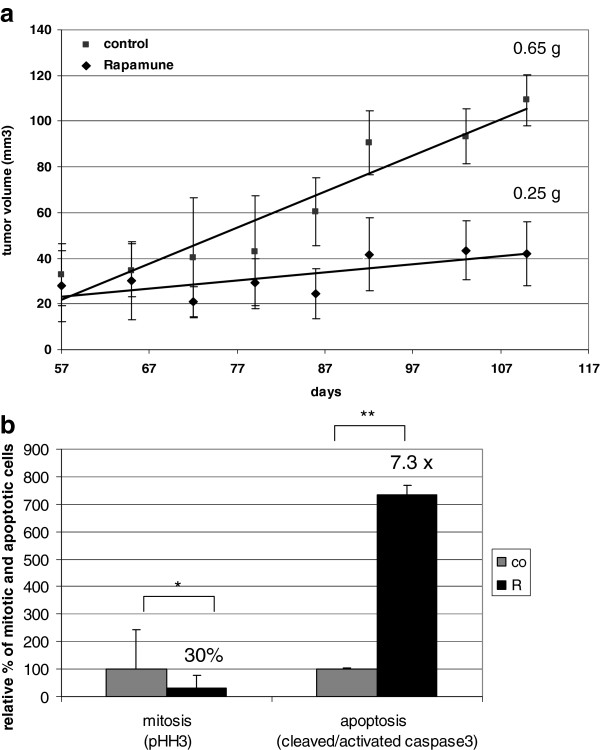
**Rapamycin inhibits tumor growth in vivo. a**. Rapamycin inhibits tumor growth in KMH2 (Hodgkin-lymphoma) xenografts in vivo. Tumor volumes are shown in control and rapamycin-treated mice during an 8-week treatment. Tumor weight at the end of the experiment is also indicated; tumors in rapamune treated mice were significantly smaller (p<0.05). **b**. Confirmation of the in vivo anti-proliferative and apoptotic effect of rapamycin by IHC detection of pHH3 (proliferation marker) and cleaved/activated caspase3 (apoptosis marker) (*:p<0.05, **:p<0.01).

The in vivo growth inhibitory effect of rapamycin was also confirmed in SCID mice with KMH2 Hodgkin-lymphoma xenografts. Rapamycin treatment (8 weeks) significantly reduced tumor volume and tumor weight in the treated animals (Figure 6a). The average tumor weight was 0.65 g vs. 0.25 g in the control vs. treated group, respectively. The significant anti-proliferative and apoptotic effect of in vivo treatment was also confirmed in KMH2 xenograft biopsies: the number of phospho-Histone H3 (pHH3; mitotic marker) positive cells were decreased (30% compared to control) and the number of cleaved/activated caspase3 (apoptotic marker) positive cells were increased (7.3× compared to control) in treated tumors (Figure 6b).

## Discussion

The introduction of new drugs has to be based on convincing evidence in malignancies where clinical response rate (or even cure rate) is rather high. A typical example is Hodgkin-lymphoma (HL); in fact, no new drugs have been approved by the FDA for HL in the last 30 years [22]. However, treatment failures in patients with advanced disease, insufficient response (recurrences and resistance) as well as late toxicity of the currently used chemotherapy – including second malignancies, cardiovascular toxicity and infertility – requires improvement in standard options for treating HL [23]. Targeted therapy is an innovative research field in oncology, where the defects of major regulatory steps fine-tuning critical cell functions such as survival, proliferation and apoptosis serve as molecular targets.

There is substantial evidence highlighting the importance of changes in the activity of different PI3K pathway members, including mTOR complexes. Here we show that mTOR activity is a characteristic feature in the majority (>50%) of MCL, BL, DLBCL, ALCL and HL cases. High mTOR activity of HRS cells is further supported by our second TMA study focusing on HLs.

Previous publications reported only small numbers of cases without considering subclassification of HL [24,25]. Based on the evaluation of different downstream mTOR target proteins in 83 HL cases, increased mTOR activity was confirmed in more than 90% of HLs in our work, which was independent of HL subtype and clinical parameters. Low mTOR activity cases had no relapse, and these patients had more than 5 year disease free survival, with complete remission. However, high mTOR activity was observed in the case of both favorable and unfavorable clinical response, therefore it cannot be considered as a prognostic indicator. We are aware that the 83 HL patients included in our study comprise a heterogeneous patient group in respect of age, gender, stage, histological type and prognosis. Therefore, it is difficult to reach significant conclusions; nevertheless, our study offers a comprehensive overview of this heterogeneous group, which is obviously characterized by high mTOR activity in general.

At a molecular level, mTOR activity is known to play a role in cyclin D1 overexpression and cell cycle dysregulation in MCL [14]. Through the regulation of translation or by directly influencing the activity of p70S6K, mTOR can induce the antiapoptotic functions of mitochondrial proteins, e.g. by BAD phosphorylation, supporting the survival and proliferation of tumor cells [26]. The malfunction of apoptotic pathways and the overexpression of several cyclins (cyclin A, B1 and E) are also known in HL [27]. The overexpression of antiapoptotic signals (Bcl-xL) showed correlation with high mTOR activity in our study.

Each time a protein known to be a member of regulatory signaling pathways, participating in the development and/or progression of malignancies is brought into focus, the question arises: can we turn our knowledge to therapeutic advantage? In the case of mTOR, inhibitors already exist (rapamycin and its analogs: rapalogs), which are well tolerated [28], and rapamycin has also been shown to synergize with anticancer agents in several tumors [12,29-31]. Rapalogs/rapamycin inhibited proliferation and induced apoptosis, moreover, they increased the apoptotic effect of chemotherapeutic agents (doxorubicin, vincristine and etoposide) in HL cells in our xenograft and in vitro experiments. These results – along with others [32-35] – suggest that mTOR inhibition is an option in tumors with increased mTOR activity. In this respect HL could be a good candidate, as high mTOR activity and mTORC1 expression could be detected in a high percentage of cases, and mTORC1 inhibition also had an antiproliferative and apoptotic effect in vitro and in vivo.

The efficiency of mTOR inhibitors may be dependent on the ratio of mTOR complexes [36]. While mTORC1 is sensitive to currently used mTOR inhibitors, the rapalog sensitivity of mTORC2 is still conflicting, and may vary in different cell types [37,38]. New dual inhibitors – inhibiting both mTOR complexes, or mTORC1 and upstream elements of the PI3K/Akt/mTOR pathway – are being developed [39]. The inclusion of upstream proteins is quite logical, because the inhibition of mTORC1 may be able to activate them. The immunohistochemical detection of the phosphorylated forms of Akt (specifically, Ser473, which is connected to mTORC2) is very difficult. We tested different antibodies but we could not detect realiably specific staining in our lymphoid tissues. Baker et al. investigated the stability of phosphorylated Akt and they established that postoperative surgical samples may be of limited value for measuring phospho-Akt levels because Akt can be dephosphorylated quickly during tumor removal and fixation [40]. Considering this, we chose to investigate the expression of Rictor, one essential component of functioning mTORC2. We concluded that mTORC2 was not a characteristic feature when Rictor expression was not detected in the samples. Several solid and lymphoid malignancies such as non-GC DLBCLs overexpress Rictor (a characteristic protein in mTORC2), which potentially indicates increased mTORC2 activity [21,41,42]. Rictor was not overexpressed in our HL cell lines and cases, which can explain the sensitivity to rapamycin/rapalogs.

Taken together, Hodgkin-lymphoma is characterized by high mTOR activity, and this high mTOR activity does not exclude good prognosis. Moreover, mTORC1 may be a potential therapeutic target in HL, especially when commonly used protocols prove ineffective, and may also allow dose reduction of chemotherapeutic drugs in order to decrease late toxicity without diminishing treatment efficacy. The combination of mTOR inhibitors with other agents targeting critical molecular sites will likely be crucial for achieving the best clinical response.

## Conclusion

Based on our results, mTOR activity may be a potential therapeutic tool in different lymphoma types. In particular, the majority of Hodgkin-lymphomas have high mTOR activity (with no mTORC2/Rictor expression). These data, along with our in vitro and in vivo results with mTOR inhibitors suggest that the inhibition of mTORC1 may be feasible in the therapy, especially in Hodgkin-lymphomas when standard protocols prove ineffective. The combination of mTOR inhibitors with other agents will probably offer the highest efficiency for achieving the best clinical response, and may also allow dose reduction in order to decrease late treatment toxicity in these cases.

## Abbreviations

ALCL: Anaplastic large-cell lymphoma; BL: Burkitt-lymphoma; cHL: Classical Hodgkin-lymphoma; CLL: Chronic lymphoid leukemia/small lymphocytic lymphoma; CR: Complete remission; DLBCL: Diffuse large B-cell lymphoma; HE: Hematoxylin-eosin; HL: Hodgkin-lymphoma; HRS cell: Hodgkin-/Reed-Sternberg cell; HSCT: Hematopoietic stem cell transplantation; LD: Lymphocyte depleted; LR: Lymphocyte rich; MC: Mixed cellularity; MCL: Mantle cell lymphoma; mTOR: Mammalian target of rapamycin; mTORC: mTOR complex; NLPHL: Nodular lymphocyte predominant; NS: Nodular sclerosis; pHH3: phospho-Histone H3; PI3K: Phosphatidylinositol-3-kinase; pmTOR: phoshpo-mTOR; p70S6K: p70S6 kinase; pp70S6K: Phospho-p70S6 kinase; pS6: Phospho-S6; p4EBP1: phospho-4EBP1; SCID: Severe combined immunodeficiency; S6K: S6 kinase; TG: Treatment group; TMA: Tissue microarray; 4EBP1: Eukaryotic translation initiating factor 4E-binding protein1.

## Competing interests

The authors declare that they have no competing interests.

## Authors’ contributions

AS was the principal investigator, designed the study, supervised materials, data collection and analysis, and takes primary responsibility for the paper. ÁM, NN and TBS designed and prepared TMA blocks and performed IHC stainings and statistical analysis for this study. MH, LB, LK, JCs took part in the morphological evaluation of sections and evaluated IHC results. ÁM, NN and VV performed in vitro and in vivo experiments, MCs and ZsV collected clinical data for the study and participated in analysis. AS, MH, ÁM and KL wrote the paper. All authors read and approved the final manuscript.

## Pre-publication history

The pre-publication history for this paper can be accessed here:

http://www.biomedcentral.com/1471-2407/13/250/prepub
